# Comment on “The Impact of Intradialytic Exercise on Activities of Daily Living and Physical Function in Hospitalized Hemodialysis Patients: A Study of Efficacy and Safety”

**DOI:** 10.31662/jmaj.2026-0139

**Published:** 2026-07-24

**Authors:** Tomoya Hirai

**Affiliations:** 1 Division of Rehabilitation, Department of Clinical Practice and Support, Hiroshima University Hospital, Hiroshima, Japan

**Keywords:** intradialytic exercise, rehabilitation, hemodialysis, hospitalized patient

## Dear Editor,

I read with great interest the recent article by Takahashi et al. ^(1)^ titled “The Impact of Intradialytic Exercise on Activities of Daily Living and Physical Function in Hospitalized Hemodialysis Patients.” The authors should be commended for addressing an important clinical problem: rehabilitation time is often reduced on hemodialysis (HD) days, and strategies to preserve function in hospitalized HD patients are clearly needed. This study is also valuable because it adds inpatient data to a field in which most prior exercise- and intradialytic exercise (IDE)-related evidence has been generated among people receiving maintenance hemodialysis, predominantly in outpatient settings ^(2), (3)^.

However, several issues merit clarification. First, the observed benefit may reflect increased rehabilitation exposure on HD days rather than an IDE-specific effect. Rehabilitation time differed mainly on HD days, whereas the IDE program itself consisted largely of supervised bed-based stretching and resistance exercise. A time-matched comparator, such as bedside exercise delivered after HD or on non-HD days, would have helped distinguish the effect of the intradialytic setting itself from the effect of simply increasing exercise opportunities.

Second, rehabilitation exposure would have been more informatively presented on a session basis rather than as minutes per hospital day. The protocol delivered rehabilitation six days per week, excluding Sundays, and discontinuation was permitted because of contraindications, physician judgment, or patient request. Yet the manuscript does not report the numbers of planned and completed conventional rehabilitation sessions on HD or non-HD days, nor the numbers of planned and completed IDE sessions, interruption frequency, cancellations, or reasons for non-completion. Without these data, treatment fidelity and the actual delivered rehabilitation dose remain difficult to interpret.

Third, it remains unclear what criteria were used to define safety in practice. Safety assessment appears to have relied mainly on summary hemodynamic measures and intradialytic hypotension frequency. However, averaged blood pressure and heart rate values do not show whether clinically relevant instability occurred in individual sessions. Likewise, without reporting interrupted, shortened, or cancelled sessions and the reasons for them, it is difficult to determine whether IDE was truly well tolerated or simply delivered selectively under favorable conditions.

Overall, this study provides useful preliminary insight into the possibility of securing rehabilitation time on HD days. Clarification of session-based exposure, comparator choice, and session-level safety would strengthen the manuscript and improve its clinical interpretability.

## Article Information

### Author Contributions

Contributed to the conception of this work, drafting and revising the manuscript, and final approval of the version to be published: Tomoya Hirai.

### Conflicts of Interest

None

### IRB Approval Code and Name of the Institution

Not applicable

### Data Availability Statement

No new data were generated for this manuscript.

### AI Declaration

A generative AI tool was used for language editing in the preparation of this manuscript
